# Inter-hospital transport of critically ill patients; expect surprises

**DOI:** 10.1186/cc11191

**Published:** 2012-02-12

**Authors:** Joep M Droogh, Marije Smit, Jakob Hut, Ronald de Vos, Jack J M Ligtenberg, Jan G Zijlstra

**Affiliations:** 1 Department of Critical Care, University Medical Center Groningen, University of Groningen, Hanzeplein 1, 9700 RB Groningen, The Netherlands; 2 Department of Technical Support, University Medical Center Groningen, University of Groningen, Hanzeplein 1, 9700 RB Groningen, The Netherlands; 3 Department of Anaesthesiology, University Medical Center Groningen, University of Groningen, Hanzeplein 1, 9700 RB Groningen, The Netherlands; 4 Department of Internal Medicine, University Medical Center Groningen, University of Groningen, Hanzeplein 1, 9700 RB Groningen, The Netherlands

## Abstract

**Introduction:**

Inter-hospital transport of critically ill patients is increasing. When performed by specialized retrieval teams there are less adverse events compared to transport by ambulance. These transports are performed with technical equipment also used in an Intensive Care Unit (ICU). As a consequence technical problems may arise and have to be dealt with on the road. In this study, all technical problems encountered while transporting patients with our mobile intensive care unit service (MICU) were evaluated.

**Methods:**

From March 2009 until August 2011 all transports were reviewed for technical problems. The cause, solution and, where relevant, its influence on protocol were stated.

**Results:**

In this period of 30 months, 353 patients were transported. In total 55 technical problems were encountered. We provide examples of how they influenced transport and how they may be resolved.

**Conclusion:**

The use of technical equipment is part of intensive care medicine. Wherever this kind of equipment is used, technical problems will occur. During inter-hospital transports, without extra personnel or technical assistance, the transport team is dependent on its own ability to resolve these problems. Therefore, we emphasize the importance of having some technical understanding of the equipment used and the importance of training to anticipate, prevent and resolve technical problems. Being an outstanding intensivist on the ICU does not necessarily mean being qualified for transporting the critically ill as well. Although these are lessons derived from inter-hospital transport, they may also apply to intra-hospital transport.

## Introduction

Transport of critically ill patients is increasing. Establishment of regional ICU centers, centralization of certain surgical procedures and availability of certain therapeutic interventions, in addition to logistic problems, causes an increase in inter-hospital transports. Intra-hospital transport frequency increases because of new imaging modes and radiological intervention modes that cannot yet be performed bedside. Both intra-hospital and inter-hospital transports pose a serious threat for patient safety [1-3]. Research has proven the value of specialized transport teams [4-6]. Teams of physicians and ICU nurses are trained to work in narrow ambulances, have learned to deal with specific transport-related medical problems and have knowledge about the technical limitations of transport. Many articles have been published about patient care and medical problems during transport [1,3,4,7,8]. Technical issues are less often addressed.

Recently, we described our first experiences with a mobile intensive care unit (MICU) and compared our results to transport with standard ambulances [3,5]. Although the population transported by MICU was severely ill with a significantly higher Acute Physiology and Chronic Health Evaluation II (APACHE II) score, there were fewer adverse events than during standard ambulance transports. However, the percentage of transports complicated by adverse events was still 12.5%, all caused by technical failures [5]. Fortunately, there was little impact on patient health.

To obtain a better understanding about the scope, relevance and kind of technical problems, we retrospectively studied all technical problems encountered during a period of two and a half years. In this article we present the results of this study and lessons that were learnt.

Knowledge of these problems may improve the quality of transport in critically ill patients not only between hospitals, but also within a hospital.

## Materials and methods

Our trolley contains all the equipment necessary for transportation: a ventilator, syringe pumps, a suction unit, monitor and defibrillator. This equipment is checked for functionality and battery status every day.

A digital transport form is completed for all transport. This contains a section about transport related events. Furthermore, a log is kept on a daily base. All events, small or major, transport-related or diagnosed at the daily equipment checks, are written down in this log.

We retrospectively analyzed all transports and all the logs for technical problems from March 2009 until August 2011. The causes and the solution of all problems were determined. If relevant, lessons learnt and their influence on protocol were stated as well.

## Results

**Figure 1:**
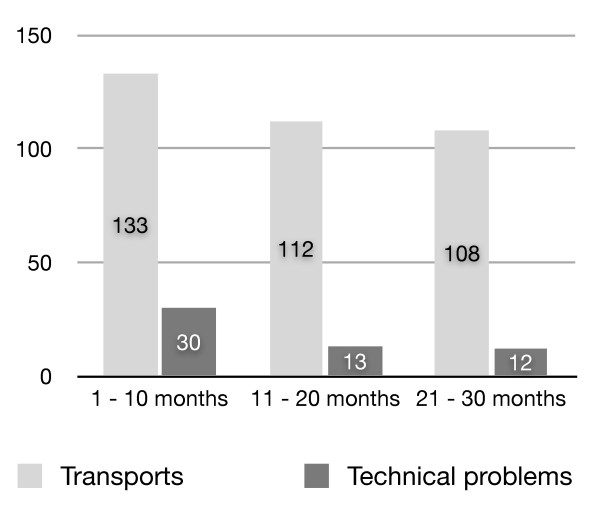
**Number of transports and technical problems in the first, second and third ten months**.

During the study period 353 patients were transported. In total 55 problems were encountered. Most of the problems occurred in the beginning of our service (Figure 1). In Table 1 we present all technical problems we encountered. Problems varied from small to serious. Although there was little impact on patient status, some transports had to be postponed, cancelled or referred. Modifying an ambulance to a MICU puts high demands on the technical properties of the vehicle and makes it more prone to technical failures. The background, solution or impact of some problems is relevant for everyone involved in transporting patients. We would like to give some examples.

**Table 1:** Technical related events.

System		Number of events
Gas supply	leakages	3
	dysfunctional gas tube connectors	5
	defective connectors of 2 liter tanks	3
	unintentional use of 2 liter tanks	3
Electrical system	defective dynamo	2
	blown fuses	4
MICU ambulance	starting problems	1
	dysfunctional loading bridge	3
	dysfunctional exterior lights	2
	collision	1
	dysfunctional heating/air conditioning	2
	minor defects on doors	4
	defective suspension	1
Equipment	dysfunctional monitor	1
	dysfunctional perfusor pump	1
	defective battery ventilator	1
	defective battery defibrillator	1
Trolley	dysfunctional brake system (blown fuses)	6
	damaged bolting system trolley	4
	collision damage on trolley or equipment	7

### The power supply

The trolley for patient transport has a huge electric power supply. It is equipped with six syringe pumps (Alaris ^® ^GH, Cardinal Health) with an internal battery with a minimal battery life of 4 hours. The trolley has a Philips ^® ^IntelliVue MP50 monitor with an internal battery capacity of at least 5 hours. The built-in ICU ventilator, Dräger ^® ^Evita XL is extended with two external batteries to last at least 2 hours. Our trolley has pressurized brakes because of its weight of almost 600 kg. Pressure is built by an electronic motor, powered by an internal battery within the trolley. Furthermore, a Laerdal ^® ^suction unit and a Philips ^® ^defibrillator are also connected to the central power supply cable of the trolley. All electronic equipment on the trolley is provided with 230 volt (AC) 50 Hz from MICU wall outlets. Subsequently its batteries will be charged and external power is used preferentially. Before the MICU is allowed to leave, a checklist has to be completed. This checklist contains a section to check if batteries are charging when the central power supply cable of the trolley is plugged into the wall outlet of the MICU ambulance.

On several occasions devices were not charging when the trolley's central electric power cable was plugged into the wall outlet. This was caused by blown fuses in the MICU ambulance due to a high peak-point current. Although the fuses could handle the basal power requirement, peak currents occurred during connection or start up, causing the fuse to blow. By changing the fuse to one more capable of dealing with a higher peak-point current, this problem was resolved.

The peak power of the trolley is sufficient to blow fuses even in the hospital environment. Connection of the trolley to the hospital wall outlet has resulted in power loss of the hospital equipment around the patient's bed on one occasion. The trolley is now preferably connected to the high power outlet reserved for radiology equipment.

### The gas supply

The Evita XL ventilator requires compressed air and oxygen. Our trolley has a storage capacity of three times two liter 200 bar tanks of both gases. A connecting tube between the trolley gas system and gas wall outlets in hospitals can be used. This connection is also used for the gas supply during transport in the MICU. Our MICU contains four 10 liter 200 bar tanks of both compressed air and oxygen. They pressurize the gas wall outlets in the MICU. Ideally the gas supply with the largest capacity is used first: the wall outlets are used in the hospital, the 10 liter tanks during transport in the MICU and the 2 liter tanks for transport on the trolley between the ICU and the MICU.

**Figure 2:**
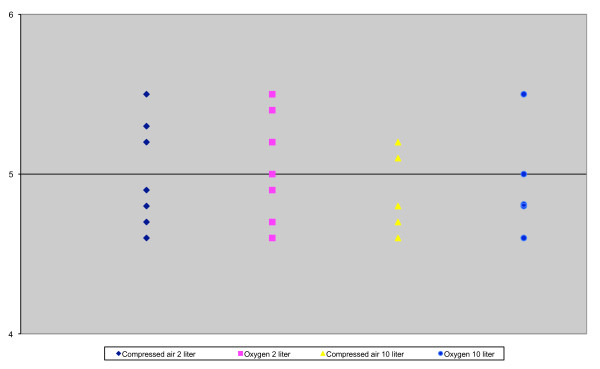
**Variations of pressure, measured at the pressure reducer, in different tanks (Bar)**.

On several occasions, we noticed a rapid decline in pressure in the two liter tanks of the trolley. Leakages were excluded. Finally we realized it was caused by using both trolley and MICU tanks simultaneously. Each bottle is connected with the gas system of the trolley with a pressure reducer with a fixed outlet pressure of 4.5 bar. There are no valves in the system. However, a random selection of two and ten liter tanks showed pressures under static conditions between 4.5 and 5.5 bar (Figure 2). Although this is quite a big range, it is still in line with official regulations which accept a range from 3.5 to 5.6 bar. To make it even more complicated not all bottles and pressure reducers have the same pressure response under dynamic conditions. As a consequence, when multiple bottles are connected it is unpredictable which will be emptied first and the trolley tanks may be drained without notice.

As a safety measure, we changed our protocol. Nowadays as soon as the central gas tubes are connected to a wall outlet in hospital or in the MICU, the two liter tanks are closed. As a byproduct of our measurements we became aware of a gas consumption of around 3.6 liters per minute of the Evita XL. Although this is stated in the manual, this characteristic is not usually recognized. It is used as a driving force and adds up to the minute volume. The unnoticed gas consumption of the ventilator can easily reach 30% of the total gas consumption and therefore limits the radius of action of the trolley and MICU.

### The vehicle

Traffic participation is a risk factor of its own that can be limited but not eliminated by experienced ambulance drivers. While transporting a critically ill patient, our MICU was hit by a 4 × 4 vehicle causing serious damage to the MICU but without casualties. The MICU could still drive, although slowly. The decision was made to continue to the hospital of destination and deliver the patient. However, this example shows the importance of having a back-up plan in case the MICU is not able to drive further. The disadvantage of having a specially designed ambulance and trolley is the difficulty of replacing it in an accident. Although we did not have to use it in this case, there is a back-up plan in case of an accident.

## Discussion

This inventory of events illustrates that despite thorough development and testing, technical incidents will occur. These technical problems occurred mostly in the first few months after we started using our custom made trolley and ambulance. Although the problems can be related to the new equipment they illustrate the importance and impact of technical failures which have the potential to occur long after start up of a retrieval service. These events are more common where specialist teams are not available [9].

Most of these events will not occur in our ICUs. Even if they did, because of the number of personnel, availability of technical support and spare equipment, they would rarely have serious consequences. On the other hand, intra-hospital transport is also transport with potential risks [1]. Lessons taken from events during inter-hospital transport may also be applied to intra-hospital transport. Furthermore, these lessons may be transferrable to other areas of medical transport as well, since problems with battery charge and gas supply have previously been identified in other retrieval services [10].

The dependence on equipment and the absence of support during transport indicates the importance of a certain technical understanding of the equipment that is used. The technical equipment used during transport surmounts the experience of most intensivists and ICU nurses, especially when problems occur. Specialized retrieval teams have proven to be of value in inter-hospital patient transport [4,5], not only because of their transport experience, and in that regard their preparation of a transport, or their knowledge of specific transport-related medical problems, but also because of their knowledge of transport related technical problems that may arise. Intensivists and critical care nurses whose work is restricted to the ICU and who are not familiar with transport may underestimate the importance of these limitations. In terms of crew resource management it might very well be that not every health care worker used to working in the highly controlled surroundings of an ICU has the mental skills to perform this challenging job.

These examples show that all systems that can fail will fail. Fortunately, most eventualities can be anticipated and, therefore, can be trained. Simulation training to achieve special skills is known to be valuable [11,12]. Team training, especially crew resource management (CRM) training is thought to be important as well [13-15]. All our MICU personnel participate in scenario-based CRM simulation trainings. They take place in an ICU environment in the skills lab as well as in the MICU itself. Medical skills, teamwork and communication are included in the training, with emphasis on the aspects specific to transport: general transport preparation, preparation of the patient, how to anticipate medical and technical problems that may arise during transport and how these may be resolved. The breakdown of all three major systems, that is, the power supply, the gas supply and the vehicle are incorporated into our simulation training scenarios.

## Conclusion

In conjunction with our previous study, the events described in this article emphasize that MICU transport is a specialty with its own demands. This has major implications for selection and training of personnel. Transport of critically ill patients is like an expedition. Participants have to be selected, preparation should be excellent, training extensive and surprises expected.

## Key messages

- When using technical equipment, technical problems may occur and should be anticipated

- For safe transport, simulation training should not only focus on medical problems but also on technical problems and their resolution.

- A certain understanding of the equipment used may be of vital importance for resolving technical problems, especially in situations with little support, such as transporting patients

## Abbreviations

CRM: Crew Resource Management; ICU: Intensive Care Unit; MICU: Mobile Intensive Care Unit

## Competing interests

The authors declare that they have no competing interests.

## Authors' contributions

JD set up the design of the study, performed data acquisition, carried out data analysis and drafted the manuscript. MS helped with acquisition of data and revised the manuscript. JH participated in the design of the study and helped with resolving and clarifying the technical problems. RV helped with interpretation of the data and revised the manuscript. JL participated in the design of the study and revised the manuscript. JZ revised the manuscript and has given final approval of the version to be published. All authors have read and approved the manuscript for publication.
